# Exploring healthy and climate-friendly diets for Danish adults: an optimization study using quadratic programming

**DOI:** 10.3389/fnut.2023.1158257

**Published:** 2023-06-15

**Authors:** Matilda Nordman, Anne Dahl Lassen, Anders Stockmarr, Pieter van ‘t Veer, Sander Biesbroek, Ellen Trolle

**Affiliations:** ^1^National Food Institute, Technical University of Denmark, Kgs. Lyngby, Denmark; ^2^Department of Applied Mathematics and Computer Science, Technical University of Denmark, Kgs. Lyngby, Denmark; ^3^Division of Human Nutrition and Health, Wageningen University and Research, Wageningen, Netherlands

**Keywords:** sustainable diets, quadratic programming (QP), diet optimization, greenhouse gas emission, dietary intake, dietary guidelines

## Abstract

**Background:**

A transition to healthy and sustainable diets has the potential to improve human and planetary health but diets need to meet requirements for nutritional adequacy, health, environmental targets, and be acceptable to consumers.

**Objective:**

The objective of this study was to derive a nutritionally adequate and healthy diet that has the least deviation possible from the average observed diet of Danish adults while aiming for a greenhouse gas emission (GHGE) reduction of 31%, corresponding to the GHGE level of the Danish plant-rich diet, which lays the foundation for the current healthy and sustainable food-based dietary guidelines (FBDGs) in Denmark.

**Methods:**

With an objective function minimizing the departure from the average observed diet of Danish adults, four diet optimizations were run using quadratic programming, with different combinations of diet constraints: (1) nutrients only (*Nutri*), (2) nutrients and health-based targets for food amounts (*NutriHealth*), (3) GHGE only (*GHGE*), and finally, (4) combined nutrient, health and GHGE constraints (*NutriHealthGHGE*).

**Results:**

The GHGE of the four optimized diets were 3.93 kg CO_2_-eq (*Nutri*), 3.77 kg CO_2_-eq (*NutriHealth*) and 3.01 kg CO_2_-eq (*GHGE and NutriHealthGHGE*), compared to 4.37 kg CO_2_-eq in the observed diet. The proportion of energy from animal-based foods was 21%–25% in the optimized diets compared to 34% in the observed diet and 18% in the Danish plant-rich diet. Moreover, compared to the average Danish diet, the *NutriHealthGHGE* diet contained more grains and starches (44 E% vs. 28 E%), nuts (+230%), fatty fish (+89%), eggs (+47%); less cheese (−73%), animal-based fats (−76%), total meat (−42%); and very limited amounts of ruminant meat, soft drinks, and alcoholic beverages (all-90%), while the amounts of legumes and seeds were unchanged. On average, the mathematically optimized *NutriHealthGHGE* diet showed a smaller deviation from the average Danish diet compared to the Danish plant-rich diet (38% vs. 169%, respectively).

**Conclusion:**

The final optimized diet presented in this study represents an alternative way of composing a nutritionally adequate and healthy diet that has the same estimated GHGE as a diet consistent with the climate-friendly FBDGs in Denmark. As this optimized diet may be more acceptable for some consumers, it might help to facilitate the transition toward more healthy and sustainable diets in the Danish population.

## 1. Introduction

Anthropogenic activities have led to the transgression of several planetary boundaries defined as the safe operating space for humanity with respect to the earth’s biogeophysical limits (1). The environmental impacts of food production are especially profound, with global agriculture being the main driver of biodiversity loss, disruption of nitrogen and phosphorous cycles, land-system change, and freshwater use (2, 3). In addition, the global food system is responsible for approximately one third of anthropogenic greenhouse gas emissions (GHGE) (4). While contemporary food production and consumption are unsustainable from an environmental point of view, they can also be regarded as such from a health perspective. Suboptimal diets—be it over-or underconsumption of energy, nutrients, and foods—are a major driver of the disease burden globally (5). Diets lie at the intersection between human health and environmental sustainability, and a transition toward sustainable healthy diets has the potential to improve both (6, 7).

Sustainable healthy diets encompass many different dimensions and are defined by the Food and Agricultural Organization (FAO) and the World Health Organization (WHO) as “dietary patterns that promote all dimensions of individuals’ health and wellbeing; have low environmental pressure and impact; are accessible, affordable, safe and equitable; and are culturally acceptable” (7). Both synergies and trade-offs exist between the different dimensions of sustainable healthy diets. Several studies have reported existing co-benefits of diets on health and environment (8, 9), and especially a transition to more plant-based diets has been identified as a promising path toward the synergistic benefits to health and environment (3, 10). On the other hand, a lower environmental burden of a diet does not guarantee its healthiness, and conversely, a healthy diet is not necessarily environmentally sustainable (11–13). In addition, several diet modeling studies have demonstrated that while diet-related GHGE can be substantially reduced, while simultaneously ensuring nutritional adequacy, the required dietary shifts may be very large and diet acceptability compromised (14, 15).

In 2019, the EAT Lancet commission on Healthy Diets from Sustainable Food Systems presented a global sustainable and healthy reference diet (3). This diet integrates scientific targets for healthy diets and sustainable food systems to create a global healthy reference diet within planetary boundaries. Using scenario analysis, Lassen et al. later modeled a national adaptation of the EAT Lancet reference diet (the Danish plant-rich diet), which lays the foundation for the Danish climate-friendly food-based dietary guidelines (FBDGs) (16, 17). The Danish plant-rich diet integrates nutrient recommendations for the reference age group 6–65 years with health-based food recommendations from reviews of epidemiological studies. The amounts of foods in the plant-rich diet are within the food group intake ranges of the EAT Lancet planetary reference diet. Compared to the average Danish diet, the Danish plant-rich diet is characterized by limited amounts of meat (especially ruminant meat), animal-based fats, and discretionary foods; moderate amounts of dairy and vegetable oils; and increased amounts of fruit, vegetables, legumes, nuts, seeds, and fish (16). Trolle et al. demonstrated that a transition among Danish adults from the average current diet to the Danish plant-rich diet would result in a 31% carbon footprint reduction (18).

While being nutritionally adequate and having a reduced environmental impact, the Danish plant-rich diet requires major changes from the Danish population’s current diet, putting the acceptability of this diet into question. Achieving a population-wide dietary shift can be difficult, as evidenced by generally low compliance to already existing FBDGs (19). Therefore, identifying dietary patterns that have the least deviation possible from current dietary habits might be a more effective approach to formulating more acceptable diets and facilitating a dietary transition in a greater proportion of the population. At the same time, a stern focus on the nutritional adequacy, health, and environmental perspectives needs to be maintained. To tackle the multi-dimensional nature of dietary choices, mathematical optimization has proven a useful technique, as demonstrated by studies in several countries (20–22). Mathematical optimization aims to minimize or maximize a given function, known as the objective function, while fulfilling specific constraints. In diet optimization, the aim is typically to arrive at an optimal combination of foods (i.e., the decision variables) to achieve the optimal solution of the objective function, e.g., minimizing GHGE or cost, while fulfilling a set of constraints given by, e.g., nutrient recommendations or price. Given a set of nutritional and environmental constraints, a large variety of possible dietary patterns exists to fulfill them. In contrast to *a priori* scenario approaches, mathematical optimization models can, when carefully constructed, provide a more data-driven way of choosing the most appropriate diet, in addition to facilitating the fulfillment of a wide range of diet constraints (23).

The objective of this study was to derive a nutritionally adequate, healthy, and low-GHGE diet that has the least deviation possible from the observed average diet of Danish adults. Quadratic programming was applied to achieve a diet with the same GHGE as the Danish plant-rich diet (31% reduction), to show an alternative way of composing a nutritionally adequate, healthy, and climate-friendly diet. Additionally, the influence of different combinations of constraints was studied to observe trade-offs and synergies between different diet dimensions and demonstrate the impact of different constraints on the resulting optimized diet.

## 2. Methods

### 2.1. Dietary intake data

Dietary intake data from the Danish National Survey of Diet and Physical activity (DANSDA) 2011–2013 was used for diet optimization in the present study. DANSDA 2011–2013 is the latest national dietary survey in Denmark, surveying a representative sample of 4-75-year-old Danes on dietary habits using a 7-day pre-coded food diary. Household measures and portion size pictures are used to estimate portion sizes, and consumed food intakes are interpreted through standard recipes into food composition table items in the Danish food composition database (24). DANSDA and the methods of dietary intake data collection are described in detail elsewhere (25). In the present study, we used dietary intake data from 2,492 adults (51.8% women) aged 18–64 years (mean age 42.8 years). To standardize all individuals regardless of gender to the same energy intake level and enable comparability of the observed diet with the optimized diets, food intakes were proportionally adjusted to a total individual energy intake of 10 MJ (2,390 kcal), corresponding approximately to the daily reference energy requirement of an average adult (across sex and age at a moderate physical activity level) (26).

In total, intakes of 434 food composition table items represent the average diet in the selected age group in DANSDA, including products in both raw/uncooked (e.g., apples and flour) and cooked/processed state (e.g., sausages and bread). Food items were aggregated into 50 food sub-groups based on nutritional, culinary, and environmental impact characteristics. The food sub-groups represent the decision variables in the optimization models, i.e., the variables whose quantities should be determined in an optimal way. Food sub-groups, rather than single food items, were used as decision variables to ensure greater diversity in food intake, maintain easy communication of the results and to account for uncertainties in nutrient composition and GHGE data of single food items. Food sub-groups were further aggregated into main food groups for the reporting of results. Food sub-groups and main groups are described in Supplementary Table S1. Food quantities in the Danish plant-rich diet were aggregated into the same main food groups and food sub-groups to enable comparison between diets.

Data on the nutritional content of foods was obtained from the newest version of the Danish food composition database (24). The content of nutrients in all food sub-groups were weighted based on the average consumption of the single food items in the population, as described previously by Gazan et al. (20):


(1) $$A_{ij}=\overset{n_{j}}{\underset{k=1}{\sum }}\frac{x_{kj}}{\sum_{k=1}^{n_{j}}x_{kj}}\times a_{ik}\;$$

where $A_{ij}$ is the content of nutrient *i* per gram of food sub-group *j*; $n_{j}$ is the number of food items belonging to food sub-group *j*; $x_{kj}$ is the quantity of food item *k* of food sub-group *j* consumed in the population and $a_{ik}$ is the content of nutrient *i* in food item *k*.

### 2.2. Greenhouse gas emission data

Similarly to nutritional content, the weighted GHGE values for each of the 50 food sub-groups were calculated based on the GHGE associated to single food composition table items. GHGE values were obtained from previous work by Trolle et al., who compiled GHGE of foods on the Danish market (18). These data are based on Life Cycle Assessment (LCA) studies in existing literature in combination with standard factors for emissions from downstream activities, such as processing, transportation, and cooking (18). The GHGE value of each food item represents the emissions from primary production (farming), processing, packaging, transport, storage, and cooking of the food item. In addition, the added GHGE from food losses are accounted for, both unavoidable food losses in the form of inedible parts (e.g., peels and bones) and avoidable food losses throughout the production chain and in retail.

### 2.3. Diet optimization

#### 2.3.1. Optimization model

Quadratic programming was applied to model diets that were as similar as possible to the observed (current) Danish diet, while fulfilling constraints for nutritional adequacy, health and environmental sustainability. Following the example of previous studies (27–29), it was assumed that the smaller the changes from the observed diet, the more acceptable the diet would be. In order to penalize large relative dietary changes, the objective of the optimization model was to minimize the quadratic relative difference from the observed average diet of Danish adults. The objective function, which was to be minimized, can be expressed as:


(2) $$\overset{50}{\underset{j=1}{\sum }}{\left(\frac{x_{j}-x_{\mathrm{obs},j}}{x_{\mathrm{obs},j}}\right)}^{2}$$

where $x_{j}$ and $x_{\mathrm{obs},j}$ are the population average amounts of food sub-group *j* in grams in the optimized and the observed diet, respectively.

The optimizations were performed using the IBM CPLEX solver implemented through the Rcplex package version 0.3–5 of the R statistical software version 4.1.3 (30).

#### 2.3.2. Constraints

To demonstrate the impact of different constraints on the resulting diet, four optimizations were performed with different combinations of constraints: *Nutri*, only nutritional adequacy constraints; *NutriHealth*, nutritional adequacy and health-based targets for food amounts; *GHGE*, only GHGE constraint; and finally, *NutriHealthGHGE*, the combined diet model with nutritional adequacy, health and GHGE constraints. An overview of the constraints applied in the models is presented in Table 1. All diets were optimized to a total energy content of 10 MJ (2,390 kcal) to enable comparison with the plant-rich diet, which was also modeled at the 10 MJ level. In addition to the nutritional constraints applied, several more nutrients were calculated in the observed and optimized diets (Table 1).

**Table 1:** Overview of variables applied as constraints in the optimization models (C) or measured (but not constrained) in optimized diets (m) and nutritional composition of the average observed diet.

			Variable applied as constraint in the model (C) or measured (m) in diet			
	Constraint limit^1^	Observed diet (per 10 MJ)^2^	Nutri	Nutri-Health	GHGE	Nutri-HealthGHGE
Energy, MJ	10	10	C	C	C	C
Protein, E%	15	16	C	C	m	C
Carbohydrates, E%	52–53	47	m	m	m	m
Added sugar, E%	≤10	9	C	C	m	C
Dietary fiber, g	≥30	**24**	C	C	m	C
Fat, E%	32–33	38	C	C	m	C
Saturated fat, E%	≤10	**14**	C	C	m	C
n-3 fatty acids, E%	≥1	1.1	C	C	m	C
MUFA, E%	10–20	13	m	m	m	m
PUFA, E%	5–10	6	m	m	m	m
ALA, E%	≥0.5	0.8	m	m	m	m
Vitamin A, RE μg	≥800	1,425	C	C	m	C
Vitamin E, alfa-TE	≥9	10	C	C	m	C
Thiamine, mg	≥1.2	1.6	C	C	m	C
Riboflavin, mg	≥1.4	1.9	C	C	m	C
Niacin, NE	≥16	38	C	C	m	C
Vitamin B6, mg	≥1.3	1.9	C	C	m	C
Folate, μg	≥450	**425**	C	C	m	C
Vitamin B12, μg	≥2	7	C	C	m	C
Vitamin C, mg	≥80	137	C	C	m	C
Vitamin D, μg	≥14	**4**	m	m	m	m
Sodium, mg	≤2,400	**3,770**	m	m	m	m
Potassium, mg	≥3,500	3,719	C	C	m	C
Calcium, mg	≥1,000	1,149	C	C	m	C
Magnesium, mg	≥320	388	C	C	m	C
Phosphorous, mg	≥800	1,580	C	C	m	C
Iron, mg	≥11.4 ^3^	**11.3**	C	C	m	C
Zinc, mg	≥9	12	C	C	m	C
Iodine, μg	≥170	198	C	C	m	C
Selenium, μg	≥57	**55**	C	C	m	C
Alcohol, g	≤17.1^4^	14.9	C	C	m	C
GHGE, kg CO_2_-eq	≤3.01^5^	**4.37**	m	m	C	C
Health-based constraints for food amounts						
Fruit, g	≥300	**243**	m	C	m	C
Vegetables, g	≥300	**226**	m	C	m	C
Fatty fish, cooked, g	≥29	**15**	m	C	m	C
Whole grain, g	≥75	**58**	m	C	m	C
Nuts, g	≥20	**6**	m	C	m	C
Red meat, cooked, g	≤50	**114**	m	C	m	C
Realism constraints						
All food subgroups, upper limit	≤90th percentile		C	C	C	C
All food subgroup, lower limit	≥0.1 × observed		C	C	C	C
Water, coffee, tea, and spices	Observed amount		C	C	C	C

Bold numbers indicate suboptimal amounts in the observed diet. Optimization models: *Nutri*, only nutritional constraints; *NutriHealth*, nutritional and health constraints; *GHGE*, only GHGE as a constraint; and *NutriHealthGHGE*, all constraints combined. GHGE, greenhouse gas emissions.

1 Micro-and macronutrient limits based on nutrient recommendations from the Nordic Nutrition recommendations (26). Macronutrient limits are transformed from E% to grams assuming no energy contribution from alcohol.

2 Based on dietary intake data for adults 18–64 years from the Danish National survey of Diet and Physical activity 2011–2013 (25).

3 Based on average requirement for pre-menopausal women.

4 Based on recommendation by the Danish Health Authority of a maximum weekly consumption of alcohol for adults corresponding to 10 standard portions (120 g alcohol/week).

5 Based on GHGE for Danish plant-rich diet (16) as estimated by Trolle et al. (18).

Lower and/or upper limits for micro-and macronutrients were based on the Nordic Nutrition recommendations (26). Macronutrient recommendations (fat, protein, and indirectly carbohydrates) were based on narrow targets for dietary planning purposes. Fatty acid quality was ensured by constraining saturated and *n* − 3 fatty acids, and additionally calculating the content of ALA, PUFA and MUFA in all diets. Micronutrient limits were based on the recommended nutrient density (per MJ) established for planning of diets for populations with a heterogeneous age and sex distribution (26). These recommendations cover an age span of 6–65 years and are based on the needs of the most demanding subgroup in the population. We deviated from these recommendations in the constraints set for zinc and iron. The recommended intake of zinc is lower for adults than for children and adolescents (12 mg/10 MJ), whereby a lower limit of 9 mg/10 MJ was selected based on the recommended intake (RI) for adults. There is a large difference in the recommendation of iron for pre-and postmenopausal women and men, with pre-menopausal women having a substantially higher iron requirement due to iron losses associated to menstruation (26). Initial optimizations indicated a difficulty in fulfilling the high iron recommendations of pre-menopausal women without imposing large changes in the diet, and therefore, the average requirement (AR) of pre-menopausal women, which was estimated to 11.4 mg/10 MJ, was selected as the constraint in the optimizations. To demonstrate the large impact of a higher iron recommendation on the optimization outcome, and to meet the requirements of most women, the optimizations were also run with a higher (16 mg/10 MJ) iron amount, corresponding to the RI for pre-menopausal women. This is further detailed in the discussion section. Vitamin D requirements can partly be satisfied by exposure of the skin to sunlight (26), and during winter months supplementation is recommended (31). Therefore, dietary vitamin D content was merely observed, rather than constrained, in the models.

The health-based constraints for food amounts included upper or lower boundaries based on epidemiological evidence on the association between health and intake of the food. The constraints are consistent with the health-based recommendations behind the Danish FBDGs. We did not include constraints on foods that are recommended as part of a healthy diet due to nutrient content and/or food culture, but only on foods for which the epidemiological evidence associating intake to disease outcome is solid. The health-based constraints for foods included in the models were: whole grain ≥ 75 g per day (32), fatty fish ≥ 200 g cooked fish per week (33), red meat ≤ 350 g cooked meat per week (33, 34), nuts ≥ 20 g per day (33, 35), and fruit and vegetables ≥ 300 g each per day (33). Since the limits for meat and fish were based on cooked amounts, the raw amounts in the dietary intake data were assigned preparation factors [0.8 for fish and 0.75 for meat (36)] and the corresponding prepared weight equivalents per day were calculated. The whole grain content in all grain products was estimated as the proportion of the whole grain ingredient of the food’s total weight based on previous studies (32), and the whole grain constraint was subsequently applied on the sum of whole grain in the diet.

The GHGE of the diet was constrained to at most 3.01 kg CO_2_-eq (31% decrease compared to baseline), corresponding to the GHGE of the Danish plant-rich diet, which is recommended as a sustainable healthy diet according to the Danish FBDGs (17, 18).

To prevent the optimization models from including unreasonably high amounts of any single food sub-group, or completely eliminating others, “realism” constraints were applied to all models, using an approach similar to that of Chaudhary and Krishna (37). An upper limit was set for food sub-groups corresponding to the 90th percentile of the observed intake among consumers in the population and a lower limit dictating that the optimized amount of a food sub-group cannot be lower than 0.1 times the average observed amount. In addition, water, coffee and tea, and spices were fixed to baseline level due to their secondary role in the diet and to maintain the same prerequisites for the optimized diets as for the Danish plant-rich diet. As a consequence of spices (including discretionary salt) being fixed to baseline level, sodium was not constrained, but rather calculated in the rest of the diet (excluding spices) and compared to recommendation.

By studying the values of the dual variables, it was determined which nutrient constraints were limiting in the diet (the active constraints), i.e., the fulfillment of the constraint had an impact on the foods selected in the diet. Furthermore, the dual values were used to determine the comparative strength of each constraint, i.e., most difficult constraints to meet.

### 2.4. Diet similarity

As a proxy for acceptability, a diet departure score (Δ_diet_) was used to estimate the similarity between the average observed diet and optimized diets, and for comparison, the Danish plant-rich diet. The departure score was represented by the average relative deviation from the observed diet (across food sub-groups), and was calculated by


(3) $$\Delta_{diet}\left(\%\right)=\frac{1}{50}\overset{50}{\underset{j=1}{\sum }}\left|\frac{x_{j}-x_{obs,j}}{x_{obs,j}}\right|\times 100$$

where $x_{j}$ is the amount of food sub-group *j* in the optimized diet or the Danish plant-rich diet and $x_{\mathrm{obs},j}$ is the average observed amount of food sub-group *j*.

## 3. Results

### 3.1. Food contents in the optimized diets

The food amounts in the observed diet and results of the diet optimizations are shown in Table 2 for main food groups and full results for all 50 food sub-groups in Supplementary Table S2. Overall, the contribution of energy from animal-based foods was lower in all optimized diets (21–25 E%) compared to the observed diet (43 E%; Supplementary Table S3). The relatively lower contribution of animal-based foods catered to the fulfillment of both nutritional and GHGE constraints. All optimized diets contained higher amounts of grains and starches, and a lower amount of cheese (Table 2). A relatively high content of dairy products (especially milk and fermented dairy products) was generally preserved in the optimized diets. Nutritionally optimized diets contained substantially less discretionary foods and animal-based fats in favor of plant-based fats, whereas in diet optimizations where a GHGE constraint was imposed, meat from ruminants, high-GHGE fish, and “other cheese” (mainly hard cheeses) were significantly reduced (Supplementary Table S2).

**Table 2:** Content of main foods groups (g) and GHGE (kg CO_2_-eq) per 10 MJ in the average observed diet, the Danish plant-rich diet, and optimized diets.

			Optimized diet			
	Observed diet^1^	Danish plant-rich diet^2^	Nutri	Nutri-Health	GHGE	Nutri-Health-GHGE
GHGE	4.37	3.01	3.93	3.77	3.01	3.01
Grains and starches						
Wheat bread	86	97	105	110	112	160
Rye bread	64	143	109	91	84	103
Pasta, rice, cereals^3^	60	71	68	68	69	83
Potatoes	85	100	98	95	98	99
Vegetables and fruit						
Vegetables	226	307	257	300	204	300
Fruit and berries	243	303	263	300	223	300
Dairy foods						
Milk	273	213	310	328	202	312
Other dairy	47	38	38	40	44	40
Cheese	45	20	11	29	14	12
Protein sources						
Beef and lamb^3^	51	9	39	19	5	5
Pork^4^	88	10	87	43	58	58
Poultry^3^	29	38	32	43	19	35
Egg	25	16	31	31	23	37
Fish and shellfish^4^	36	63	43	54	30	46
Legumes^3^	1	40	1	1	1	1
Nuts and seeds	6	38	9	20	7	20
Meat and dairy substitutes	1	1	1	1	1	1
Fats						
Fat, plant-based	28	27	39	32	42	34
Fat, animal-based	12	4	1	2	12	3
Discretionary foods and beverages						
Discretionary foods	58	19	30	30	83	26
Soft drinks	190	54	207	179	164	19
Alcoholic beverages	241	75	237	220	162	24
Water, spices, and stimulants						
Water, coffee, and tea	1,987	1,946	1,987	1,987	1,987	1,987
Spices and miscellaneous	22	22	22	22	22	22

GHGE, greenhouse gas emissions.

1 Based on dietary intake data for adults 18–64 y from the Danish National survey of Diet and Physical activity 2011–2013 (25).

2 Diet model laying the foundation for the Danish food-based dietary guidelines (16).

3 Product in raw/dry weight.

4 Mix of raw and processed products (e.g., ham, sausage, smoked, and canned fish).

The most notable differences between the observed diet and the optimized *NutriHealthGHGE* diet were considerable relative increases in the amounts of nuts (+230%), bread (+75%), and eggs (+47%); near elimination of ruminant meat, soft drinks, and alcoholic beverages (all −90%); and decreases in animal-based fats (−76%), cheese (−73%), discretionary foods (−55%), and pork (−34%). The decrease in pork came especially from a decrease in processed pork products (e.g., sausages and hams). The total amount of fish and shellfish was moderately increased (+28%) but the types of fish were redistributed to substantially less high-GHGE fish (e.g., plaice and shrimp) in favor of increased amounts of fatty fish (e.g., mackerel, herring, and salmon). While total amounts of fruit and vegetables were increased (+24% and + 33%, respectively), these increases were especially large for coarse vegetables and pome fruit. Overall, the energy contribution from grains and starches increased from 28 E% in the observed diet to 44 E% in the optimized *NutriHealthGHGE* diet, while discretionary energy decreased from 18 E% to 5 E% (Supplementary Table S3).

**Figure 1:**
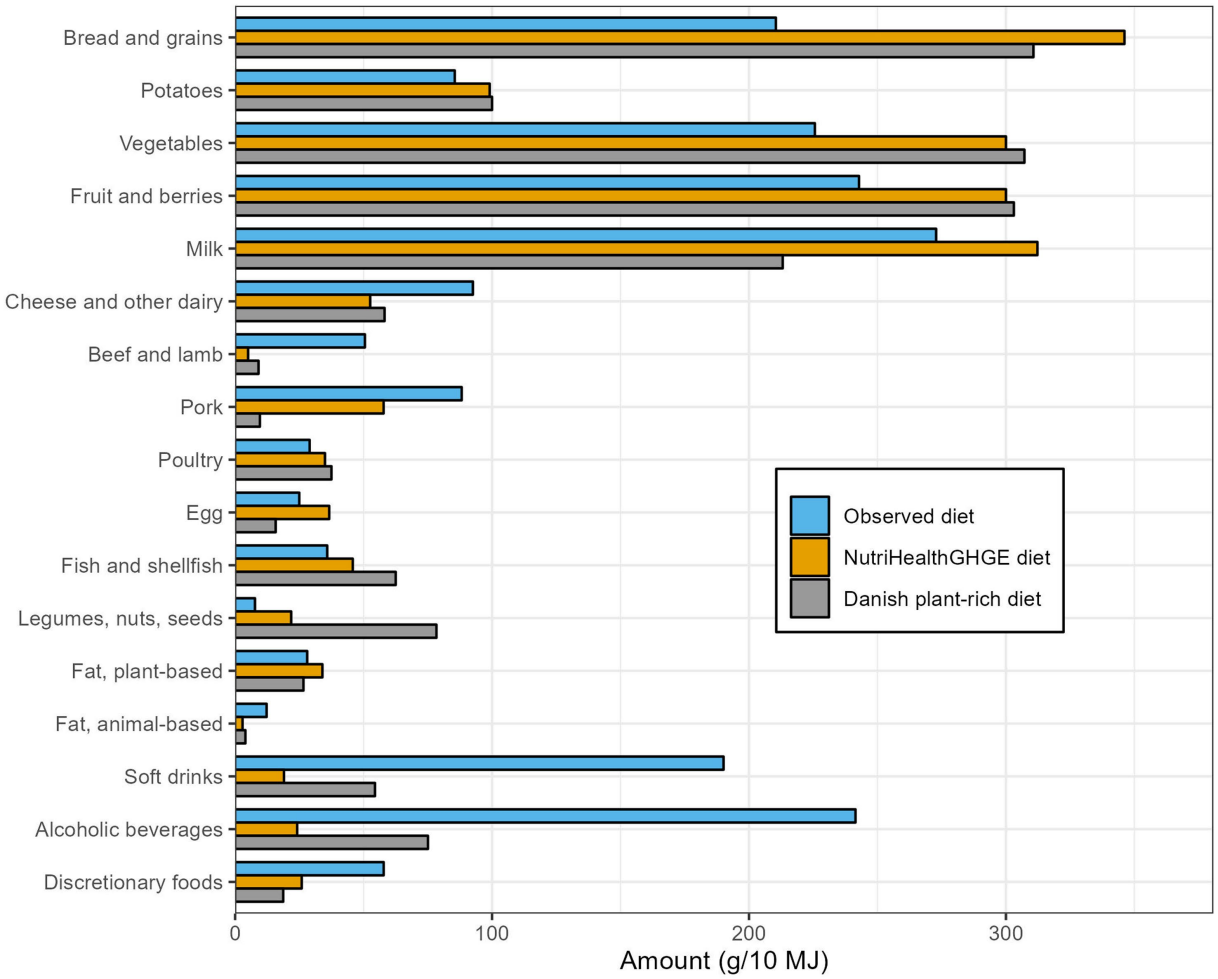
Comparison between average observed diet among Danish adults, the optimized *NutriHealthGHGE* diet, and the Danish plant-rich diet, which lays the foundation for current climate-friendly food-based dietary guidelines in Denmark. All food amounts standardized to total energy intake of 10 MJ.

When comparing the *NutriHealthGHGE* diet with the Danish plant-rich diet, differences were observed especially in the protein sources of the diets (Table 2; Figure 1). The total amount of meat in the optimized *NutriHealthGHGE* was 98 g compared to 56 g in the Danish plant-rich diet, with the largest share of the meat being pork in the optimized diet and chicken in the plant-rich diet. To meet the same GHGE reduction goal, the *NutriHealthGHGE* diet had a lower content of especially ruminant meat, high-GHGE fish, soft drinks, and alcoholic beverages, which all hit the lower boundary set by the realism constraints (Supplementary Table S2). The proportion of energy from all animal-based foods was 22 E% in the optimized diet and slightly lower (18 E%) in the plant-rich diet. The plant-rich diet contains in total more than three times more legumes, nuts, and seeds and 36% more fish, while the *NutriHealthGHGE* diet contains six times more pork, more than twice the amount of eggs, and 50% more milk compared to the plant-rich diet. Although the amounts of vegetables were increased in both diets, the Danish plant-rich diet promotes comparably a larger increase in dark green vegetables (16), while different types of vegetables are increased in the *NutriHealthGHGE* diet.

When the diet was constrained for higher dietary iron to meet the recommendation for pre-menopausal women (*NutriHealthGHGE-Iron*), more substantial increases in specific nutrient-rich carbohydrate sources (especially coarse wheat bread, rye bread, oats, and potatoes) were observed compared to the optimization with a lower iron constraint (Supplementary Table S4). In addition, this diet contained higher amounts of pork offal and eggs, while ruminant meat, fatty dairy products, cheese, high-GHGE fish, animal-based fats, soft drinks, alcoholic beverages, and discretionary foods were decreased all the way to the lower boundary set in the optimization (Supplementary Table S4).

### 3.2. Nutritional contents in the optimized diets

The average observed diet contained suboptimal amounts of dietary fiber, saturated fat, folate, vitamin D, sodium, iron, and selenium compared to target amounts (Table 1). The macronutrient distribution did not meet the narrow targets for planning of diets for populations but was within acceptable ranges for individuals (26).

The nutritional contents of the optimized diets are shown in Supplementary Table S5. Limiting nutrients in nutritionally constrained diet models varied between optimized diets but included fat and protein (in all nutritionally optimized diets due to narrow targets), dietary fiber (in *Nutri* diet), saturated fat (in *Nutri* and *NutriHealth* diets), calcium (in *Nutri* and *NutriHealthGHGE* diets), and selenium (in *Nutri* and *NutriHealth* diets). The *GHGE* model (which had no nutritional constraints) did not fulfill the requirements for many micro-and macronutrients and health-based food targets. All optimized diets contained a higher amount of sodium than recommended. This is partly due to the decision to maintain spices (including discretionary salt) at level of the observed diet and not constrain sodium content. The content of sodium excluding sodium from spices was between 2.3 g (in *GHGE* diet) and 2.5 g (in *Nutri* and *NutriHealthGHGE* diet). Content of vitamin D was far below recommendation in all optimized diets but higher than in the average observed diet.

Iron content was 12.0–12.5 mg in the optimized diets, except in the *GHGE* model where iron was 10.8 mg (Supplementary Table S5). In all optimizations where the higher iron constraint value was used, the dual values indicated that iron was by far the most limiting constraint (data not shown), and therefore, the strongest determinant of the dietary changes required from the observed diet.

### 3.3. GHGE and diet similarity

The GHGE of the observed diet was 4.37 kg CO_2_-eq (Table 2). The *Nutri* and *NutriHealth* models were not constrained for GHGE but had nevertheless lower GHGE than the observed diet: 3.93 kg CO_2_-eq (−10%) and 3.77 kg CO_2_-eq (−14%), respectively. The GHGE of the *Nutri-Iron* and *NutriHealth-Iron* diets were 4.54 (+4%) and 3.74 (−15%), respectively (data not shown).

The diet departure scores for the optimized diets were 17% for the *Nutri* diet, 27% for the *NutriHealth* diet, 16% for the *GHGE* diet, and finally, 38% for the *NutriHealthGHGE* diet. The slightly higher diet departure scores of the *NutriHealth* and *NutriHealthGHGE* diets were mainly driven by a relatively large increase in nuts from baseline (from 6 to 20 g). For comparison, the diet departure score of the Danish plant-rich diet was 169%. The high diet departure score of the Danish plant-rich diet was mainly driven by very large relative changes from the observed diet in a few food sub-groups, i.e., legumes, seeds, nuts, and dark green vegetables (16). The *NutriHealthGHGE-Iron* diet with a higher constraint limit for iron had a diet departure score of 73%.

## 4. Discussion

### 4.1. Main results

All mathematically optimized diets implemented in the present study to obtain climate reduction alone (*GHGE* diet), nutritional adequacy alone (*Nutri*) or combined with health-based targets for food amounts (*NutriHealth*) and climate reduction (*NutriHealthGHGE*) contained less animal-based foods in favor of more plant-based foods compared to the average observed diet among Danes. More specifically, to meet the GHGE reduction goal of 31%, as well as nutritional and health targets, the optimized combined *NutriHealthGHGE* diet presented in this study contained more grains and starches, nuts, fruit and vegetables, plant-based fats, fatty fish, and eggs and less meat (especially beef and processed pork products), cheese, animal-based fats, high-GHGE fish, and discretionary foods and beverages compared to the average Danish diet but similar amounts of legumes and seeds.

In agreement with our findings, a recent review identified a dietary transition toward more plant-based diets as the main finding of 12 studies optimizing the health and sustainability of diets in different countries (21). Although decreased, a moderate amount of animal-based foods remained in the optimized *NutriHealthGHGE* diet but the types of animal-based foods were redistributed toward less beef and cheese, in particular, in favor of eggs, milk, and moderately more fish. Comparable patterns of redistribution of animal-based foods toward less meat in favor of dairy, egg and fish products were demonstrated in a study using a similar quadratic programming optimization approach in a Mediterranean diet at an environmental impact reduction of 30% (29).

In addition, in the *NutriHealthGHGE* diet we observed a shift in the dietary energy contribution away from animal-based foods and discretionary energy (i.e., sweet and savory snack foods, alcoholic-and sugar-sweetened beverages) to increased energy especially from grains and starches. The Danish plant-rich diet prompts a similar dietary shift (16), and an increase in grains and starches has also been observed in the majority of previous optimizations studies (38). In a recent review, Poutanen et al. highlight the importance of grains as a source of environmentally sustainable and healthy plant proteins that could play an important role in the transition to healthy diets from sustainable food systems (39).

The optimized *Nutri* and *NutriHealth* diets had a 10 and 14% lower GHGE compared to the observed diet despite not being constrained for GHGE. These results indicate that with a starting point in the observed Danish diet, focusing on nutritional adequacy and health alone will result in lower GHGE. The synergistic benefits to diet quality and GHGE were mainly driven by a decrease in high-fat animal-based foods (cheese, butter, and other high-fat dairy products), which catered to lowering the content of saturated fat and simultaneously resulted in lower GHGE. In the *Nutri-Iron* diet, however, the high iron constraint resulted in an increase in ruminant meat and a slight GHGE increase of 4%, indicating a potential trade-off between strict nutritional requirements and GHGE reduction goals. In the *NutriHealth and NutriHealth-Iron* diets, the health-based upper limit on the content of red meat additionally contributed to lower GHGE. To reduce GHGE in the GHGE-constrained optimized diets, a large reduction in ruminant meat was required, consistent with findings from previous optimization studies in several different countries (15, 21, 28, 38, 40–44). The *GHGE* model, while meeting the GHGE target of 3.01 kg CO_2_-eq, did not fulfill the requirements for many nutrients and health-based food targets (e.g., saturated fat, dietary fibers, folate, iron, calcium, and selenium). Therefore, when the observed diet is the starting point, focusing on lowering GHGE alone will not result in a healthy or nutritionally adequate diet, indicating the importance of taking nutrition into consideration when deriving low-GHGE diets.

The departure scores of the optimized diets indicate that fulfilling nutrient and health requirements of the diets alone (without any constraint for GHGE) requires relatively large departure from the observed diet, which is further increased when a limit for GHGE is introduced, indicative of a trade-off between health/GHGE and acceptability. Reducing the GHGE of the diet beyond the present 31% reduction would require larger changes in the diet and likely challenge the acceptability of changes for most consumers. Previous studies in France, the Netherlands, and the United Kingdom have found thresholds of 30–40% reduction in the environmental impact of the diet, beyond which major departure from the observed diets would be required (15, 40, 45, 46).

In general, mathematical optimization models are sensitive to the local conditions (food culture, nutrient intake, environmental footprints etc.) and assumptions made in different methodological choices (discussed further in section 4.4). This makes comparisons across studies difficult but also highlights the importance of studying nation-specific settings to create relevant dietary models and recommendations.

### 4.2. Comparison between optimized *NutriHealthGHGE* diet and Danish plant-rich diet

We aimed for a GHGE reduction of 31% from the observed diet, consistent with the 3.01 kg CO_2_-eq that Trolle et al. estimated for the Danish plant-rich diet, which lays the foundation for the healthy and sustainable FBDGs in Denmark (18). A transition from the current Danish diet to the plant-rich diet requires substantial changes in some foods (i.e., a considerable increase in especially legumes, nuts and seeds and decrease in red meat), which might compromise diet acceptability and limit the potential for a dietary transition in the population. Therefore, in this study, we optimized the diet to minimize the deviation from the current diet, in an attempt to improve diet acceptability and provide an alternative healthy and sustainable diet. In comparison to the Danish plant-rich diet, the mathematically optimized *NutriHealthGHGE* contains more meat (especially pork) and animal-based foods overall (including eggs and milk). To attain the same GHGE reduction, the *NutriHealthGHGE* diet requires relatively larger decreases in certain high-GHGE foods (cheese, ruminant meat, and high-GHGE fish) and discretionary beverages (alcoholic beverages and soft drinks) compared to the plant-rich diet. While the mathematically optimized diet outperforms the plant-rich diet in terms of similarity with the observed diet (as indicated by a lower diet departure score), the aforementioned differences between the two low-GHGE diets may have different acceptability among different individuals.

### 4.3. Nutritional aspects

In accordance with the constraints applied in the optimization, the contents of all nutrients in the *NutriHealthGHGE* diet were consistent with recommendations and planning goals from NNR, except for sodium and vitamin D. However, when discretionary sodium from the “Spices and miscellaneous” food sub-group was disregarded, the sodium content of the optimized diet nearly adhered to recommendation. Following the *NutriHealthGHGE* diet would on average require a total sodium reduction of 37%. A large part of this reduction could be achieved through decreased use of discretionary salt, but focus on food reformulation strategies to, e.g., decrease salt content in bread are also important due to the high intake of sodium from processed foods in most western countries (47).

Due to our decision to use the AR for premenopausal women as the constraint limit for iron, rather than the RI, there is a proportion of women whose iron requirements are not met with the *NutriHealthGHGE* diet. Instead, to ensure a sufficient iron intake for more than 90% of pre-menopausal women, the climate-friendly diet should be composed as the *NutriHealthGHGE-Iron* in Supplementary Table S3. This high-iron diet requires larger changes from the observed diet and may therefore have poorer acceptability as a recommended diet. These kinds of trade-offs between acceptability, nutritional adequacy, and environmental sustainability are important to consider in the planning of diets for the formulation of generalized FBDGs for a population. For example, to what extent the nutritional needs of a specific part of the population should determine the recommendations for the entire population, possibly at the expense of wider diet acceptability, and furthermore, to what extent the absolute healthiness and maximal acceptability of the diet should be ensured at the expense of potential further improvements to environmental sustainability. For individuals with higher requirements of iron (and other nutrients), other strategies to increase the intake and absorption may be considered instead, e.g., increasing the bioavailability of nutrients through cooking and meal planning practices, a relatively higher consumption of specific nutrient-rich foods (e.g., blood sausage for iron), or the consumption of fortified foods or dietary supplements. Difficulties in fulfilling iron recommendations are common in diet optimization studies. To address the problem, some have similarly to us used the AR instead of RI (48), accepted a below-recommended amount of iron in the optimized diet (43, 49), or allowed the increase of single high-iron foods (e.g., liver) by carrying out optimization on food item level (28).

Other critical micronutrients that determined the outcome of the optimization were calcium and selenium, indicating that these nutrients require special attention when deriving lower-GHGE diets. In previous studies in high income-countries, critical nutrients in diets with lower environmental impact include in addition to the aforementioned nutrients for example α-linoleic acid, retinol, fiber, saturated fatty acids, thiamin, and zinc (40, 43).

### 4.4. Methodological choices, strengths, and limitations

As the health dimension of diets encompasses more than just nutrient adequacy, a strength of the present study is our comprehensive approach to the healthiness of the diet by inclusion of both nutritional adequacy and epidemiology-based targets for food groups. Previously published optimization studies commonly describe “health” purely by nutrient adequacy without regard for the healthiness of the foods from which those nutrients are provided (37, 40, 50, 51). In addition, through the stepwise addition of constraints in the four optimization models, we can observe the impact of different constraints on the resulting diet and observe trade-offs and synergies between different diet dimensions.

An additional strength is that we consider diet acceptability by minimizing the departure from the observed diet while fulfilling criteria for health and lowered GHGE. This type of approach tends to produce more realistic results than approaches that directly minimize the environmental impact of a diet (52). Despite this, acceptability is not guaranteed. A major challenge of diet optimization is the choice of relevant criteria for diet acceptability, as highlighted by Perignon and Darmon in a recent review article (23). The choice of model relies on assumptions of what is thought to be the most acceptable diet and the most acceptable dietary changes. In the present study, the average observed diet of the Danish population is assumed to be the most acceptable diet, from which departure should be minimized. However, this population-based approach fails to account for individual variability in the underlying dietary patterns of the population and differences in the needs and preferences of various consumer groups. Individual-level optimization is one option to better capture these perspectives and several such studies exist in previous literature (41, 53–56). An optimization approach that is showing promising results for diet acceptability is the use of individuals’ diets, rather than foods, as the optimization variables (decision variables) (49, 57, 58). These optimizations preserve the interdependencies between food groups or items as they are consumed by the individuals in the population and therefore have the potential to create more realistic diets. However, the possibilities of change are limited within the realm of existing diets, i.e., the optimized diet can only get as good as that of the best individual in the target population. While individual-level approaches in general can shed light on the inter-individual variability in food consumption, they are not only computationally heavier, but the results of such optimizations can be difficult to communicate in a simple way because of the multitude of optimization results. In the formulation of population-targeted generalized FBDGs, where results need to be simplified for communicational purposes, population-based approaches suffice. The optimal choice of modeling approach therefore comes down to the specific purpose of the study.

To derive sustainable and healthy diets that minimize the departure from a reference diet, the majority of previously published studies have applied linear programming (14, 15, 40, 41, 43, 44, 50, 59–62), but in more recent years, many studies applying quadratic programming have been published (27–29, 37, 45, 63). There is no standard way of defining the minimal departure from a reference diet, and the choice of function to quantify the departure greatly impacts the type of behavior favored by the optimization model. The quadratic objective function was our preferred option because it penalizes large deviations and thereby tends to generate relatively small changes to many foods, which was assumed to result in higher perceived diet acceptability. Linear objective functions on the other hand (or non-linear functions that are transformed and solved linearly), tend to generate changes to fewer foods, but those changes tend to be larger. As stated by van Dooren in a review of diet optimization studies, “quadratic programming has advantages over linear programming when the goal is to find small changes on population level” (22).

In the present study, we standardized the objective function across food sub-groups, such that the departure from the observed diet was represented by the relative (percentage) difference from the observed to the optimized diet. This is an advantage when different foods and beverages are consumed in widely different quantities (as is often the case in whole diet optimization) and absolute changes are not comparable across food sub-groups. The limitation of this approach (in combination with the quadratic objective function) is that foods that are consumed in very small amounts in the observed diet are highly unlikely to be modified markedly by the optimization model, potentially unnecessarily limiting the opportunities of change. This is for example the case for legumes, which had an observed amount of 1 g and were not increased in the optimized diets, not because there is no place for legumes in a sustainable diet (as seen in the Danish plant-rich diet) but because of the selected modeling approach. Better results could for example be achieved if the departures for different foods would be weighted differently by the use of a relevant indicator of people’s willingness to make changes to their diets. Finding relevant weighting factors to make such improvements remain perspectives for future research. Finally, standardization by division with the baseline amount causes problems for foods that have an intake of zero at the baseline (division by zero), and therefore, adding new food items to the optimized diet requires a modified strategy.

As opposed to most previous optimization studies, we used 50 food sub-groups rather than the original 434 food items as decision variables in the optimizations. The reduced number of decision variables reduces the flexibility of the model, i.e., limits the possibilities for the model to find suitable solutions, but can make communication of the results simpler, since the results of fewer variables need to be communicated through, e.g., dietary guidelines. In addition, aggregating food items into food sub-groups guarantees a variety in the underlying food items, which is key to a healthy and acceptable diet. Allowing the optimization model enough flexibility without overcomplicating the results is a difficult balance to strike, and in the present study, there is a level of subjectivity in the grouping of foods which might be better handled with statistical methods of clustering foods into groups. This notion is further enforced by the fact that the optimization is sensitive to the observed amounts of foods in the diet due to the relative term of the objective function; in essence, sensitive to the grouping of foods. Further investigations into the best way of grouping foods and the sensitivity of the optimizations are warranted.

Another important limitation worth mentioning is that only one environmental footprint, namely GHGE, was used to evaluate sustainability of the optimized diet, while the EAT Lancet global reference diet is constructed to respect six different planetary boundaries (3). Previous research by Gephart et al. compared diets minimized for different environmental footprints and found similar dietary patterns in the resulting optimizations, stating that “there are generally synergies, rather than trade-offs, among low footprint diets” (64). Nevertheless, for a more complete evaluation of environmental sustainability, and to avoid so-called burden shifting, other environmental footprints, such as land use, water use, and nitrogen footprints, should be evaluated. For example, Vellinga et al. demonstrated that healthier diets in the Netherlands were associated with lower GHGE but higher blue water footprint, stating that these environmental footprints need to be considered in unison (65). In addition to the health and environmental dimension of sustainability, social, economic, and animal welfare concerns should be addressed to avoid unintended negative consequences of a wider food system transition.

Finally, the quality and uncertainties of both the dietary intake data and the environmental footprint data are limitations that might influence the validity of the results. GHGE data are highly sensitive to the production systems they represent and quality of the input data (both nutritional and environmental) may have important implications for the optimization results. The robustness of the results in relation to data uncertainties and possible changes in future production systems need to be further investigated and taken into account in interpretation of the results and in the evaluation of absolute sustainability aspects of diets. In addition, to suggest dietary changes that are compatible with a sustainable food system, consideration of coproduction of different foods belonging to the same production system (e.g., dairy and beef, and eggs and poultry) is necessary. For example, Kesse-Guyot et al. included coproduction links by stating that for every 1 L of milk, 10 g of beef needs to be included (63). Lastly, this study is limited by the lack of diet cost as a subject of investigation, as this might be an important factor limiting acceptability, especially in lower income socio-economic groups (66).

### 4.5. Conclusion

By applying quadratic programming in four optimization models, this paper demonstrates how a nutritionally adequate, healthy, and low-GHGE diet can be composed for the adult Danish population, while having the least deviation possible from the average observed diet, in an attempt to improve acceptability. The final optimized diet represents an alternative way of composing a nutritionally adequate and healthy diet that has the same GHGE as the Danish plant-rich diet, which lays the foundation for the FBDGs in Denmark. The presented diet deviated on average less from the observed diet than the Danish plant-rich and may be more acceptable to some individuals, therefore, having the potential to help facilitate, or act as a steppingstone in a transition toward more healthy and sustainable diets in Denmark. These findings should be interpreted into relevant dietary guidelines and supplemented with targeted public health interventions and policies to guide consumers in shifting dietary habits. Future research efforts should focus on expanding optimization modeling to include a more holistic perspective of the food system and more complete evaluation of different environmental footprints, and to better take into account the preferences and needs of different consumer groups to improve acceptability of the modeled diets.

## Data availability statement

The original contributions presented in the study are included in the article/Supplementary material, further inquiries can be directed to the corresponding author.

## Ethics statement

Ethical review and approval was not required for the study on human participants in accordance with the local legislation and institutional requirements. Written informed consent for participation was not required for this study in accordance with the national legislation and the institutional requirements.

## Author contributions

MN, AL, ET, and AS contributed to conceptualization and design of the research. MN carried out data processing and calculations, performed diet optimizations with assistance from AS, and wrote the first draft of the manuscript. All authors contributed to the article and approved the submitted version.

## Funding

This study was internally funded by the Technical University of Denmark.

## Conflict of interest

The authors declare that the research was conducted in the absence of any commercial or financial relationships that could be construed as a potential conflict of interest.

## Publisher’s note

All claims expressed in this article are solely those of the authors and do not necessarily represent those of their affiliated organizations, or those of the publisher, the editors and the reviewers. Any product that may be evaluated in this article, or claim that may be made by its manufacturer, is not guaranteed or endorsed by the publisher.
